# A reciprocal regulatory circuit between CD44 and FGFR2 via c-myc controls gastric cancer cell growth

**DOI:** 10.18632/oncotarget.8764

**Published:** 2016-04-16

**Authors:** Jihyun Park, Sun Young Kim, Ha-Jung Kim, Kyoung-Mee Kim, Eun Young Choi, Myung-Soo Kang

**Affiliations:** ^1^ Department of Health Sciences and Technology, Samsung Advanced Institute for Health Sciences and Technology (SAIHST), Sungkyunkwan University and Samsung Medical Center, Seoul 06351, Korea; ^2^ Division of Hematology-Oncology, Department of Medicine, Samsung Medical Center, Seoul 06351, Korea; ^3^ Department of Pathology & Translational Genomics, Samsung Medical Center, Seoul 06351, Korea; ^4^ Samsung Biomedical Research Institute (SBRI), Seoul 06351, Korea; ^5^ BioMembrane Plasticity Research Center (MPRC), Seoul National University College of Medicine, Seoul 03080, Korea

**Keywords:** CD44, FGFR2, c-Myc, regulation, cancer

## Abstract

Despite their suggested importance, the mechanistic roles of FGFR2 and gastric cancer stem cell (GCSC) marker CD44 remain unclear. We investigated cross talk between CD44 and FGFR2. FGFR2 and CD44 positively regulate each other's expression. While FGFR2 suppresses c-Myc transcription, CD44 activates it. c-Myc in turn augments FGFR2 transcription. CD44 knockdown (KD) depleted FGFR2 and other GCSC markers, decreased c-Myc and Sox2 expression, and suppressed tumor growth, whereas CD44 activation led to FGFR2 induction. FGFR2 KD decreased most GCSC marker expression, including CD44, but increased c-Myc and Sox2 expression and attenuated tumor growth. FGFR2 kinase inhibitor and FGFR2 neutralizing antibody decreased the CD44^+/hi^ GCSC fraction. Conversely, FGFR2 overexpression increased CD44 and accelerated tumor growth in mice. FGFR2 was co-expressed and colocalized diffusively with CD44, EpCAM, and LGR5. In contrast, phospho-FGFR2 colocalized densely with CD44, forming an aggregated signaling complex that was prevented by FGFR2 inhibition. The c-Myc KD depleted FGFR2 but not CD44. Similarly to CD44^+/hi^ phenotypes, sorted FGFR^+/hi^ cells had larger volumes, formed more tumor spheres, grew faster *in vivo* with bigger tumor mass, and expressed more CD44, EpCAM, and HER2. These findings suggest that FGFR2^+/hi^ cells have stemness properties. Moreover, *in situ* FGFR2 expression in patient-derived gastric cancer tissue correlated with tumorigenic potential in a xenograft model. In conclusion, CD44 and FGFR2 maintain stemness in gastric cancer by differentially regulating c-Myc transcription.

## INTRODUCTION

A rare subset of cancer stem cells or tumor-initiating cells are capable of dictating self-renewal, thus redirecting tumor heterogeneity and tumorigenesis [1]. Despite some controversies [2, 3], CD44, CD90 (Thy1), and EpCAM (CD326) are thought to enrich gastric cancer stem cells (GCSCs) [4–6]. CD44 is a major adhesion molecule for extracellular matrix components and is a hyaluronic acid (HA) receptor. CD44 has been implicated in leukocyte homing and activation, wound healing, cell migration, and tumor metastasis [7]. Altered CD44 expression correlates with metastasis, recurrence, and overall poor outcomes for patients [1]. Despite controversies regarding the existence of GCSCs, CD44 has been used as a marker for enriching cancer stem cells (CSC) or tumor-initiating cells in many cancer types including breast cancer, GC, pancreatic adenocarcinoma, and hepatocellular carcinoma [4]. CD44 standard (CD44s) is required for tumor growth, metastasis, and post-radiation recurrence of pancreatic xenograft tumors in mice. CD44s also upregulates stem cell self-renewal genes Nanog, Sox-2, and Rex-1 and signal transducer and activator of transcription 3 (STAT3) in hepatocellular carcinoma cells [8].

Nearly 40% of all gastric cancers (GCs) have genetic alterations in at least one of the FGFR2, KRAS, EGFR, ERBB2, and MET signaling axes and FGFR2 is the most frequently amplified component [9]. Interestingly, alteration in one axis is almost mutually exclusively dominant to other axis pathways, demonstrating the existence of five distinct GC subgroups [9]. Fibroblast growth factor receptors (FGFRs) are a family of receptor-type tyrosine kinases (RTKs). FGFR signals are involved in diverse cellular and biological processes including cell proliferation, differentiation, development, tumorigenesis, apoptotic resistance, epithelial-to-mesenchymal transition (EMT), metastasis, angiogenesis, and tissue regeneration [10–13]. Members of the FGFR family (FGFR1–4) are frequently deregulated in diverse cancers [9, 11]. Multiple FGFs bind to multiple FGFRs in a tissue-specific manner [14]. These secreted-type ligands include FGF1 (acidic FGF), FGF2 (basic), FGF3-6, FGF7 (KGF), FGF8-10, and FGF16-23 [13]. Combinatory signals of FGFs-FGFRs are transduced to the RAS-mitogen-activated protein kinase (MAPK) and phosphoinositol 3 kinase (PI3K)-AKT signaling pathways via FGFR substrate 2 (FRS2), and independently to the diacyl glycerol (DAG)-protein kinase C (PKC), and inositol trisphosphate (IP3)-Ca^2+^-releasing signaling pathways via phospholipase C gamma (PLCγ) [14]. Specifically, ligand binding to FGFR leads to heterodimerization of the FGF: FGFR complex, receptor homo dimerization, receptor auto phosphorylation, and activation of downstream signaling pathways such as PI3K-AKT, MAPK-ERK, and nuclear factor of activated T cells (NFAT) [15, 16]. FGFR2 expression is correlated with progression and poor prognosis in diverse cancers including GC [17–23]. Several FGFR inhibitors have shown remarkable antitumor effects in preclinical phases and are now in clinical trials [10].

Though FGFR2 and CD44 signals are frequently deregulated and are important in GC growth and maintenance *in vitro* and *in vivo* [10, 24–26], the cooperative roles of FGFR2 and CD44 in the context of gastric cancer stemness factors have not been studied. In this study, we assessed the cooperative role of CD44 and FGFR2 in cross regulation and GC tumor initiation. An intriguing cross talk between FGFR2 and CD44 likely maintains cancer stemness by reciprocally regulating their expression via differentially regulating c-Myc transcription.

## RESULTS

### Sorted FGFR2^+/hi^ and CD44^+/hi^ or EpCAM^+/hi^ fraction of GC cells supported tumor growth *in vitro* and *in vivo*

Using highly consistent tumorigenic cells that express CD44 or FGFR2 is critical to investigate a regulatory or cooperative role between FGFR2 and CD44 (or other markers of GCSCs) in gastric tumorigenesis. We subcutaneously implanted one million cells of each of eight GC cell lines in nude mice (n = 4–5) and monitored tumor growth. Four GC cell lines (SNU-1, SNU-16, SNU-484, and MKN45) formed tumors at 4 weeks post injection. Two of these cell lines (SNU-16 and MKN45) showed reproducible, consistent tumorigenicity (Figure S1A). The SNU-16 cell line was highly tumorigenic; subcutaneous injections of as few as one or 10 cells gave rise to progressive tumors in nude mice (Figure S1B) (Figure S2A). SNU-16-derived tumors (0.54 ± 0.33 cm^3^) were larger than MKN45-derived tumors (0.30 ± 0.16 cm^3^) (Figure S1A; Figure S2 for more detail). On the other hand, the MKN45 cell line was moderately tumorigenic and required 1000 injected cells for tumor growth in two out of five nude mice (Figure S2B, S2C). MKN45 expressed CD44, but not FGFR2. The role of FGFR2 could thus be tested in MKN45 cells by introducing FGFR2. Taken together, the SNU-16 cell line formed highly progressive tumors while MKN45-derived tumors grew slower. Therefore, the SNU-16 cell line likely contains more stem cells and may be an appropriate model cell line for testing the cooperative roles of cancer stem cell functions *in vitro* and *in vivo*. In agreement with our recent report [27], the SNU-16 cell line was highly and consistently tumorigenic in xenograft models and expressed known GCSC markers (CD44, EpCAM and FGFR2; Figure S1C). Importantly, the fluorescence-activated cell sorting (FACS) fraction of CD44^+/hi^, EpCAM^+/hi^, and particularly FGFR2^+/hi^ formed more tumor spheres *in vitro* (Figure S1D, S1E) and established larger tumor masses in mice compared to each fraction of CD44^−/low^, EpCAM^−/low^ or FGFR2^−/low)^ (Figure S1F, S1G), indicating that GCSCs are enriched in the FGFR2+, CD44+, and EpCAM+ fractions. The side population (SP)^+^ of cells did not show such increases compared to SP^−^.

### FGFR2 or CD44 depletion suppressed tumor sphere formation and tumor growth

**Figure 1:**
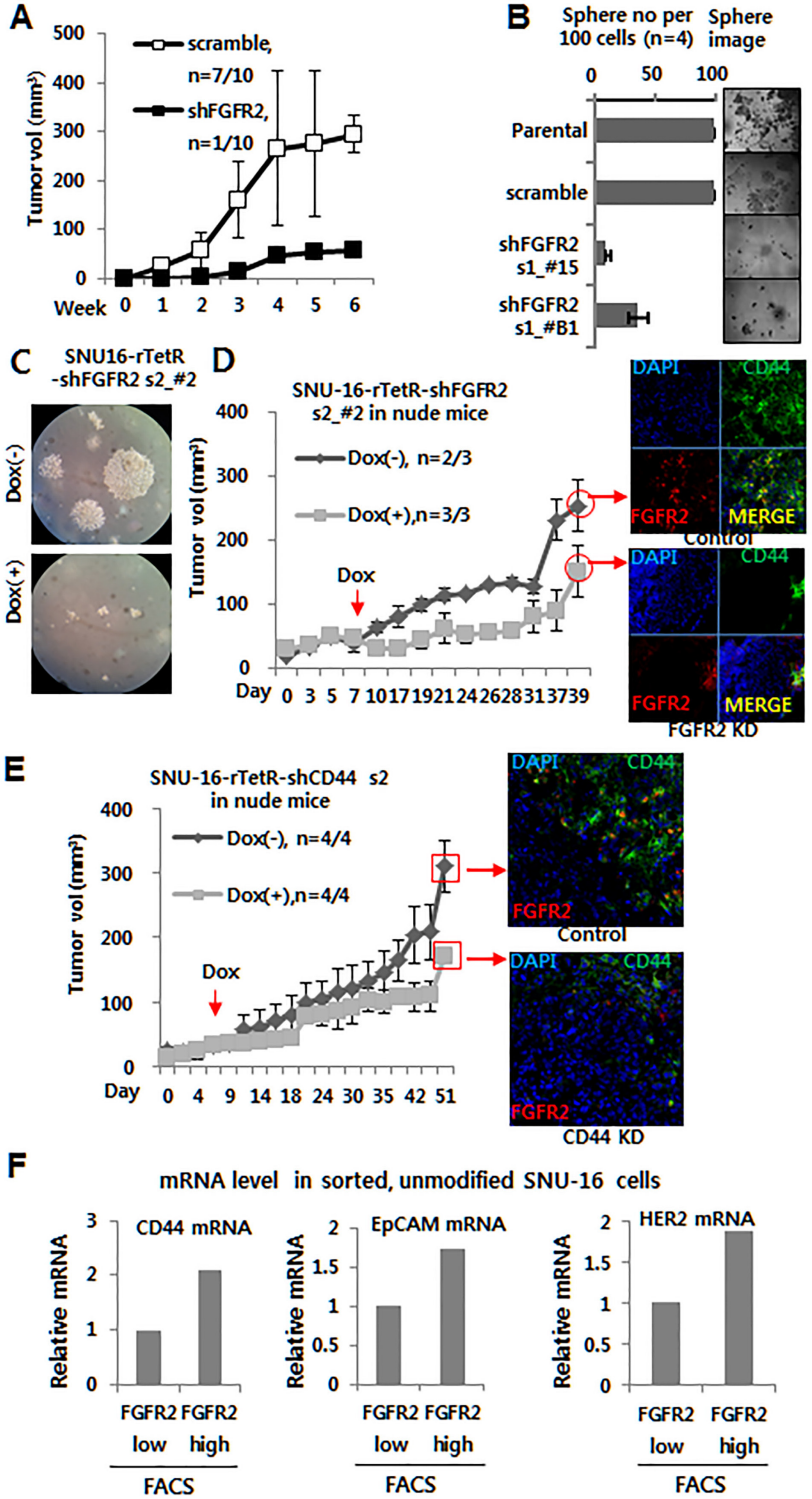
Depletion of FGFR2 or CD44 suppressed tumor sphere formation and tumor growth **A.** A shRNA s1-mediated FGFR2 KD suppressed tumor growth in nude mice (n = 10). While scrambled cells formed tumors (329 mm^3^) in seven of 10 mice, stable FGFR2 KD cells supported tumor growth in only one of 10 mice (See Figure S2 for detailed images of all 10 mice). **B.** FGFR2 KD suppressed tumor sphere formation in shRNA s1 clones (#15 and B1). **C.** Doxycycline (Dox)-induced FGFR2 KD *in vitro* suppressed colony formation in soft agar. **D–E.** Dox-induced KD of FGFR2 (D) or CD44 (E) *in vivo* suppressed tumor growth in nude mice. Dox (2 mg/ml in water) was administrated daily to mice with sizable tumors (50–100 mm^3^; n = 3). FGFR2 KD or CD44 KD in the formed tumor masses was validated by immunofluorescence (IF) staining (shown on the right). **F.** Higher levels of CD44, EpCAM, and Her2 mRNAs were found in flow cytometry-sorted FGFR2^+/hi^ fractions than FGFR2^−/lo^ fractions.

The role of FGFR2 was investigated using two small hairpin RNAs (shRNAs) specific to FGFR2 (shFGFR2 set 1 and set 2). The shRNA set 1-mediated stable knockdown (KD) of FGFR2 resulted in suppressed cell growth *in vitro* (Figure S1H), reduced tumor growth in nude mice (n = 10) (Figure 1A), and reduced tumor sphere formation *in vitro* (Figure 1B). These results were further confirmed by conditional shRNA set 2 expression, wherein shRNA expression was induced by doxycycline (Dox) *in vitro* and *in vivo*. For this, SNU-16 cells were engineered to express TetR (pLenti6/TR) (Invitrogen) and TetR-responsive, Dox-inducible FGFR2 KD shRNA set 2 vector (or CD44 vector wherever needed). Dox-induced FGFR2 KD *in vitro* attenuated colony formation in soft agar (Figure 1C). Most of all, FGFR2 KD *in vivo* suppressed tumor growth in mice as shown by multiple independent experimental replicates (Figure 1D, Figure S2D). In a reciprocal experiment (Figure S1I), Dox-induced CD44 KD *in vivo* suppressed tumor growth in nude mice (Figure 1E) and in non-obese diabetic/severe combined immuno-deficient mice with IL2R knock-out (NOD/LtSz-*scid/IL2Rγ*^null^) (referred to here as NSG mice) purchased from Jackson Laboratory (Bar Harbor, ME) (NSG) mice [28, 29]. Immunofluorescence (IF) staining verified a substantial KD of FGFR2 or CD44 following oral administration of Dox during tumor growth in tumor cell-injected mice and a subsequent delay in tumor growth (Figure 1D right and E right, respectively). Interestingly, FGFR2 KD was accompanied by a decrease in CD44 expression and CD44 KD was followed by a FGFR2 decrease (Figure 1D, 1E). In accordance with these results, the FGFR2^low^ FACS fraction showed reduced CD44 mRNA expression with decreased EpCAM and HER2 mRNA expression as determined by real time quantitative-PCR (RTq-PCR) (Figure 1F), suggestive of a possible reciprocal regulation between FGFR2 and CD44.

### Enforced FGFR2 expression supported tumor sphere and tumor formation

FGFR2-myc or a vector control was stably transfected in moderately tumorigenic CD44^+^/FGFR2^−^ MKN45 cells with slow growth kinetics in nude mice (Figure S1A; Figure S2B, S2C; Figure S3A). FGFR2-myc-expressing cells formed more tumor spheres (Figure S3B) and grew more progressive tumors in nude mice and NSG than the vector control (Figure S3C, S3D).

### FGFR2 colocalized with known GCSC markers (CD44, EpCAM), but not with HER2 or Thy1, and FGFR2 positive cells had enlarged cell volumes

**Figure 2:**
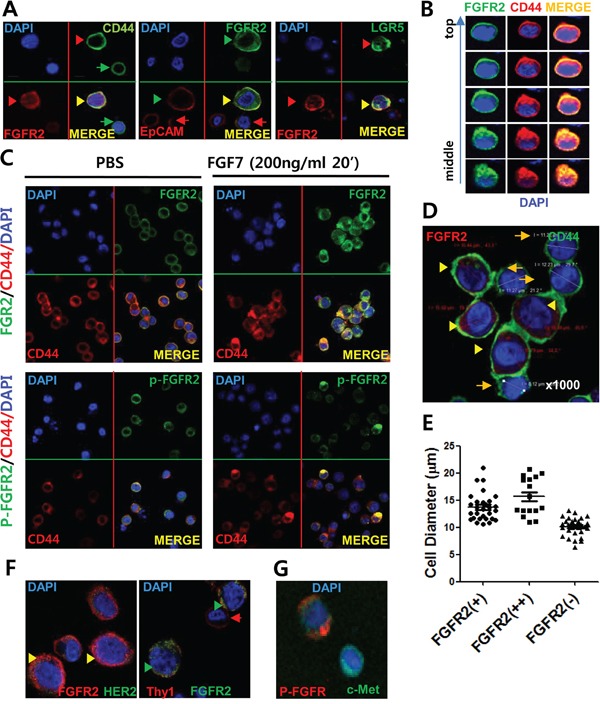
FGFR2 colocalized with known GCSC markers (CD44, EpCAM) and FGFR2 positive cells had enlarged cell volumes **A.** IF staining revealed colocalization of surface FGFR2 with GCSC markers (CD44, EpCAM) and intestinal stemness protein LGR5, but not with HER2 or Thy1. FGFR2 was expressed in both the cytoplasm and plasma membrane. Double positive cells with strong FGFR2 (FGFR2^+/hi^) and any level of CD44, EpCAM, and LGR5 expression usually displayed enlarged cell volumes (denoted with arrowhead ►) than FGFR2^−/low^ cells (denoted as arrow →) (See also Figure S4). **B.** Z stack image analyses of confocal data revealed colocalization of surface FGFR2 with surface CD44. Topology from cell top to middle equator is shown. **C.** FGF7-induced FGFR aggregates with CD44 in SNU-16 cells. Phosphorylated FGFR2 (p-FGFR2) colocalized as a punctate form in a periplasmic site in cytoplasm. (See more experimental replicates in Figure S4B, Figure 5D, and Figure 6B). **D.** Enlarged cell volume in FGFR2-expressing cells compared to FGFR2-nonexpressing cells. **E.** Average diameter of SNU-16 cells showing FGFR2 ^(++)^ (strong positive), FGFR2 ^(+)^ (positive), and FGFR2 ^(−)^ (negative) staining from IF microscopic analyses. Diameters of FGFR2^++^ (mean 15.8 μm), FGFR2^+^ (mean 14 μm), and FGFR ^(−)^ (mean ∼10 μm) cells were determined from 50 cells per fraction by confocal staining. In A to D, nuclei were visualized by DAPI (blue color). Indicated proteins with green color and red colors were visualized by antibodies conjugated with Alexa-Fluor 488 (or FITC) and Alexa-Flour 588 (or APC), respectively (See Table S1 for detail). **F.** No apparent colocalization of surface FGFR2 with HER2 or Thy1. Note low HER2 expression in this SNU-16 cell. **G.** No apparent association of punctate p-FGFR2 and diffuse EpCAM in response to FGF7.

Confocal microscopy with IF staining revealed that CD44 was located mostly on surface while FGFR2 was expressed both on the membrane surface (mFGFR2) and in the cytoplasm (cFGFR2) (Figure 2). Of note, the FGFR2 and CD44 double positive cells also expressed known GCSC markers (EpCAM, HER2) and another gastrointestinal stemness marker, LGR5. Consistent with previous findings (Figure 1F), mFGFR2 diffusively colocalized with CD44, EpCAM, and LGR5 (Figure 2A). This colocalization was confirmed by Z stack analyses (Figure 2B) and multiple independent IF staining (Figure 2D; Figure S4). Of importance, FGF7, a natural ligand of FGFR2, clearly induced this colocalization to aggregated or punctate form in multiple independent experiments in SNU-16 cells (Figure 2C) and 293T cells transiently transfected with CD44 and FGFR2-myc expression plasmids (Figure S4B). Notably, double positive cells with strong FGFR2 (FGFR2^+/hi^) expression and any of CD44, EpCAM, or LGR5 expression usually showed 1.5X larger cell diameter (3.5X larger cell volume) than FGFR2^−^ or CD44^+^ cells (Figure 2D, 2E). In most cases, FGFR2-expressing cells had larger cell volumes because they had enlarged cytoplasm spaces. Cells measurements confirmed that FGFR2-expressing cells had larger diameters than cells without FGFR2 expression (Figure 2D, 2E; Figure S4A1, S4A2), further supporting that FGFR2-expressing cells potentially harbor cancer stemness properties. There was no apparent colocalization of FGFR with HER2 or Thy1 (Figure 2F) or that of punctate p-FGFR2 with diffuse EpCAM (Figure 2G).

### FGFR2 KD decreases CD44 expression, but increases c-Myc and Sox 2 expression. CD44 KD decreases FGFR2, c-Myc, and Sox expression

**Figure 3:**
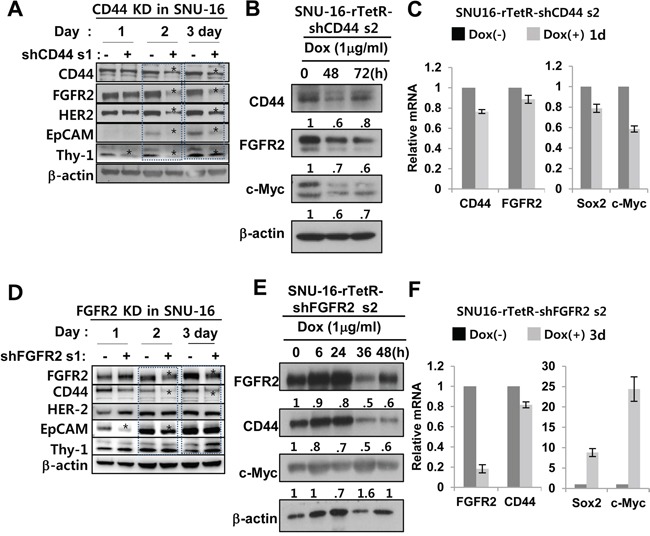
FGFR2 depletion decreases CD44 and CD44 depletion decreases FGFR2 via c-Myc **A.** A shRNA s1-mediated transient KD of CD44 decreased FGFR2, HER2, EpCAM, and Thy1 (CD90). * denotes a significant decrease. Figure S5 shows data from multiple independent experimental replicates. **B.** CD44 KD decreased c-Myc and FGFR2. **C.** Induced CD44 KD by doxycycline (Dox) for 1 day in SNU-16-TetR-shCD44 2 cells resulted in significant decreases in Sox2 and c-Myc mRNA levels but had less profound or delayed decreases on FGFR2 mRNA, indicating that CD44 regulation of FGFR2 occurs at the posttranscriptional level rather than at transcriptional level. **D.** A shRNA s1-mediated transient FGFR2 KD significantly decreased CD44 and EpCAM, but Her2 and Thy1 levels were not as significantly decreased (See Figure S5 for additional data). **E.** FGFR2 KD resulted in increased c-Myc and decreased CD44. **F.** Induced FGFR2 KD for 3 days by Dox in SNU-16-TetR-shFGFR2 set 2 was accompanied by sharp increases in Sox2 and c-Myc mRNA levels but less profound or delayed CD44 mRNA decreases. The mRNA levels were determined by RT-qPCR.

Because FGFR2 levels correlated with CD44 levels (Figure 1E, 1F), the possible reciprocal regulation between FGFR2 and CD44 was further investigated using shRNAs. Transient *in vitro* CD44 KD using shRNA decreased protein expression levels of FGFR2 and other GCSC markers (Her2, EpCAM, and Thy1 (Figure 3A). Inducible CD44 KD by Dox also decreased c-Myc and FGFR2 protein levels, but mRNA levels were decreased less (Figure 3B, 3C). FGFR2 mRNA levels were less significantly reduced by CD44 KD, but c-Myc and SOX2 mRNA levels were significantly decreased. Thus, FGFR2 regulation by CD44 likely occurs at the posttranscriptional level (Figure 3B). FGFR2 KD caused decreased CD44 levels and sharply increased c-Myc levels. I*n vitro* transient KD of FGFR2 decreased CD44 and EpCAM protein levels (Figure 3D). Inducible FGFR KD also decreased CD44 and increased c-Myc at the protein level while sharply inducing c-Myc and Sox2 mRNA expression (Figure 3E). A less significant decrease in CD44 mRNA levels was found in the same experimental set (Figure 3F). Most of these results were confirmed by multiple experimental replicates in the same cell line (two more replicates for CD44 KD and FGFR2 KD, respectively) (Figure S5A, S5B) and in a patient-derived primary GC cell line (Figure S5C) using three different shRNAs targeted to FGFR2 in the same SNU-16 cell line (Figure S5D). Alternatively, a doxycycline-dependent conditional KD of FGFR2 (Figure S6A, S6B) and CD44 (Figure S6C, S6D) was performed.

### FGFR2 activation augments CD44 signaling and CD44 activation enhances FGFR2 signaling

**Figure 4:**
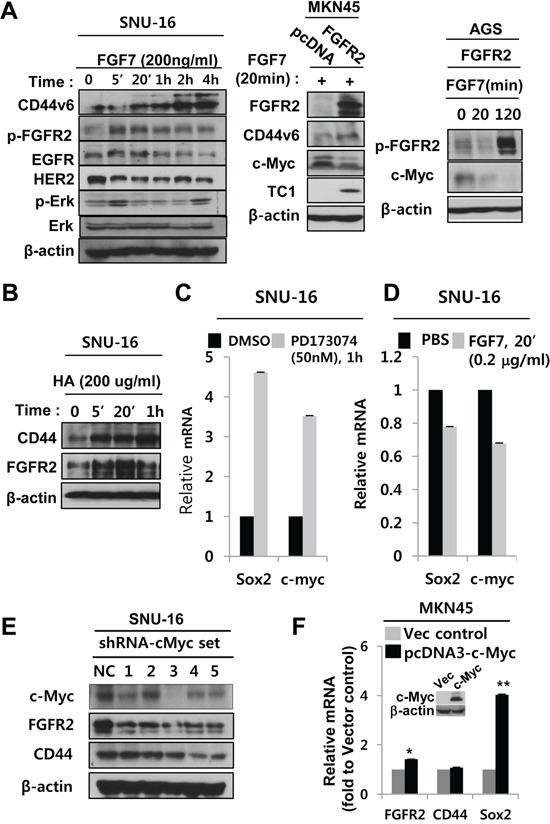
FGFR2 activation augments CD44 signaling and CD44 activation enhances FGFR2 signaling **A.** Activation of the FGFR2 signaling axis by FGF7 (left) and transfecting FGFR2 expression vector into MKN45 (middle) or AGS gastric cancer cell lines, increased CD44 but decreased c-Myc. There was no discernible change in EGFR and HER2 in multiple gastric cancer cell lines. Note concomitant transient phosphorylation of ERK, a positive control marker of FGFR2 activation. **B.** CD44 activation increased CD44 and FGFR2. **C.** FGFR2 inhibitor PD173074 enhanced Sox2 and c-Myc mRNA levels. **D.** FGF7 treatment decreased Sox2 and c-Myc mRNA. **E.** Five different shRNAs (sets 1–5) mediating c-Myc KD decreased FGFR2 levels. KD of c-Myc by shRNA sets 3–5 also decreased CD44. **F.** Transfection of human c-Myc (pcDNA3-c-Myc) into MKN45 cells increased FGFR2 (*) and Sox2 mRNA (**), but not CD44 mRNA. Western blot of c-Myc expression shown in the inset.

Positive regulation between FGFR2 and CD44 was cross-validated. Activation of the FGFR2 signaling axis by FGF7, a natural ligand of FGFR2, resulted in a transient CD44 expression increase in SNU-16 in multiple independent experimental replicates (Figure 4A left; Figure S7A). In addition, transfection of FGFR2 into the FGFR2^−/low^ CD44^low^ MKN45 GC cell line resulted in increased CD44 and thyroid cancer protein (TC1), a downstream gene of FGFR2. FGF7 caused decreased c-Myc (Figure 4A, middle; Figure S7B). Transfection of FGFR2 expression into the FGFR2^−/low^, CD44^low^ AGS GC cell line and subsequent activation by FGF7 drastically increased p-FGFR2 and decreased c-Myc, (Figure 4A right; Figure S7C), consistent with a model that c-Myc is negatively regulated by FGFR2 signaling. Protein levels of c-Myc were decreased by FGF7 (Figure 4A middle, left), validating previous results (Figure 3D, 3E). Conversely, hyaluronic acid (HA), a CD44 ligand, induced CD44 and FGFR2 expression (Figure 4B). These results were confirmed in multiple experimental replicates using all three GC cell lines (Figure S7). Following FGF7 treatment, concomitant or delayed increases or oscillating changes in phospho (p)-FGFR2 or p-ERK validated the propagation of proper FGFR2 signaling (Figure S7A). Gradual FGFR2 decreases following FGF7 treatment occurred due to transient FGFR2 internalization and/or degradation for recycling (Figure S7D). CD44 activation by HA also showed a concomitant and delayed increase in p-FGFR2 levels (Figure S7E).

### c-Myc participates in the reciprocal regulation

Consistent with Figure 3E and 3F, FGFR2 inhibitor PD173074 enhanced Sox2 and c-Myc mRNA levels (Figure 4A, 4C). On the other hand, FGF7-mediated FGFR2 activation weakly decreased Sox2 and c-Myc mRNA (Figure 4D). Since c-Myc mRNA levels were upregulated by CD44 and downregulated by FGFR2 (Figure 3B, 3D), we next investigated whether c-Myc could affect FGFR2 and CD44 levels Transient c-Myc KD by five different shRNAs to c-Myc (sets 1, 2, 3, 4, 5) (Figure 4E) and 1 siRNA (set 7) (Figure S5E) in SNU-16 cells consistently decreased FGFR2 protein levels to varying degrees, suggesting that c-Myc positively activates FGFR2 mRNA transcription. Consistence with previous results (Figure 3A, 3C), FGFR2 decrease by c-Myc KD was followed by a decrease in CD44 (Figure 4E sets 3–5). Transfection of human c-Myc into MKN45 cells increased FGFR2 and Sox2 mRNA, but not CD44 mRNA (Figure 4F).

### FGFR2 inhibition reduces the CD44^+/hi^ fraction and prevents aggregated colocalization of p-FGFR2 with CD44 *in vivo*

**Figure 5:**
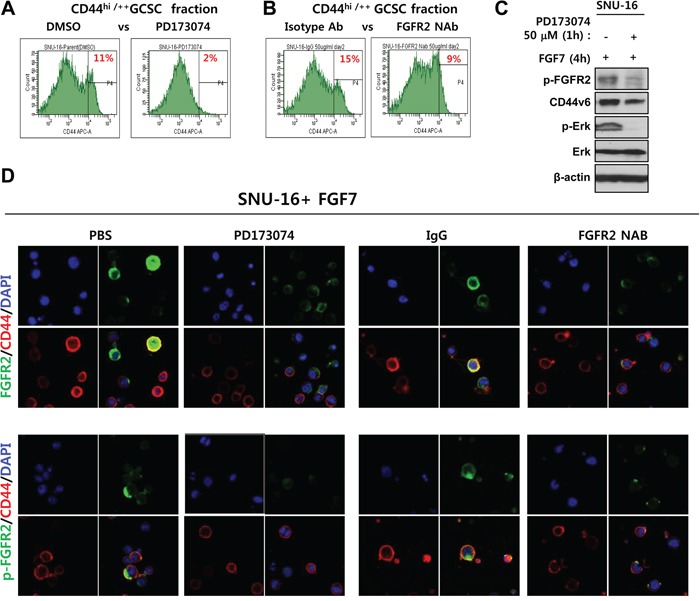
Inhibition of FGFR2 reduces the CD44+/hi fraction and prevents colocalization of FGFR2 with CD44 *in vivo* **A–B.** FGFR2 kinase inhibitor PD173074 (A) and FGFR2-blocking neutralizing antibody (NAB) (B) significantly reduced the fraction of CD44^hi/++^ potential GCSCs. **C.** PD173074 reduced p-FGFR2, p-ERK levels, and CD44 levels. **D.** PD173074 and FGFR NAB blocked CD44 colocalization with phospho-FGFR2 (p-FGFR2). Strong colocalization of bright total FGFR2 (denoted FGFR2) with CD44 was prevented by both FGFR2 inhibition methods prior to FGF7 treatment. P-FGFR2 confined to mostly periplasmic sites as a punctate form. The p-FGFR2 association with CD44 was prevented by FGFR2 inhibition.

Interplay between FGFR2 signaling and CD44 was further assessed by functional inhibition of FGFR2. The FGFR2 inhibitor PD173074 reduced the fraction of CD44^+/hi^ potential GCSCs (11% with DMSO versus 2% with PD1703074) (Figure 5A). The FGFR2 neutralizing antibody (NAB) also reduced the fraction of CD44^+/hi^ potential GCSCs (15% with an IgG control versus 9.3% with FGFR2 NAB) (Figure 5B). PD173074 also reduced CD44 protein levels (Figure 5C). Inherently constitutive or induced levels of CD44 and phosphorylated active FGFR2 in response to FGF7 were diminished by 50 nM PD173074 in SNU-16 cells (Figure 5C). In addition, functional inhibition of FGFR2 also blocked FGF7-induced colocalization of p-FGFR2 with CD44 (Figure 5D). These results indicate that FGF7-induced aggregation of p-FGFR2 with CD44 likely forms a functional intermolecular signaling complex as a punctate form in the periplasmic cytoplasm that was prevented by FGFR2 phosphorylation inhibitor PD173074 in CD44 and FGFR2 double positive SNU-16 cells.

### FGFR2 extracellular or cytoplasmic domain deletion reduced aggregated colocalization while retaining diffuse colocalization with CD44

**Figure 6:**
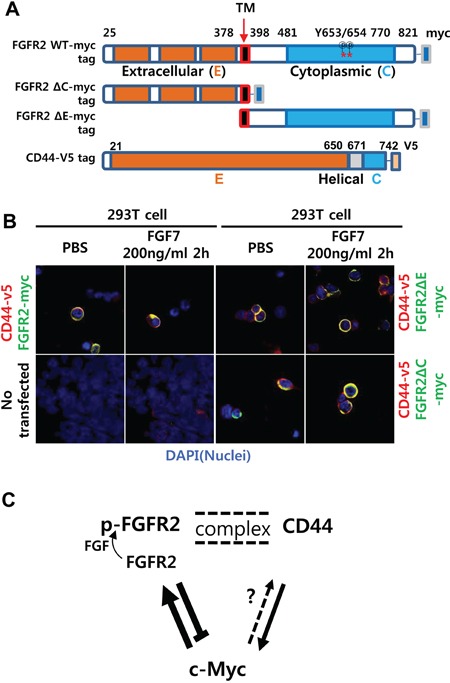
FGFR2 extracellular or cytoplasmic domain deletion reduced aggregated colocalization while retaining diffuse colocalization with CD44 **A.** Schemes of myc-tagged FGFR2IIIc (FGFR2-myc) mutants with deleted N terminal extracellular (E) or C terminal cytoplasmic (C) domains. Note there are 29 phosphorylation sites in the wild type (WT) (18 threonine/serine, 11 tyrosine). Two phospho-tyrosine sites (a. a. residues 653 and 654) recognized by the antibody used in this study are indicated with asterisks (*). **B.** Without FGF7 treatment (PBS), the full-length FGFR2 WT colocalized with CD44 in a diffuse pattern on and/or near the cell surface. With FGF7 treatment, the two molecules aggregated to a single punctate dot per cell. FGFR2 ΔE and FGFR2 ΔC showed only diffuse staining with no evident aggregates regardless of FGF7 treatment. Untransfected 293T cells were used as a negative control to validate the absence of v5 and detectable endogenous myc. **C.** A proposed model of the reciprocal regulatory circuit. Arrows (→) and bars (┴) indicate activation and repression, respectively. Double dotted lines indicate FGF7-induced formation of punctate complexes between the two proteins. Less conclusive regulation is shown as a dotted arrow. p-FGFR2 is the phosphorylated form of FGFR2.

FGF7-induced punctate colocalization of FGFR2 with CD44 likely reflects a necessity of FGFR2 phosphorylation for the association. To determine which domains of FGFR2 are necessary for this colocalization, the complete extracellular (E) domain (amino acid [a. a.] 22–377) or the intracellular cytoplasmic (C) domain (a. a. 399–821) of FGFR2 was deleted (Figure 6A). Deletion of E or C domains caused complete deficiency of FGFR2 phosphorylation at Y653 and Y654 (Figure S8). Ligand binding is necessary for receptor phosphorylation. Thus, the observed defective phosphorylation at substrate sites tyrosine 653 and tyrosine 654 in the C domain after deleting the E domain that encompasses the ligand-binding domain was expected. Obviously, deleting the C domain that contains the phosphorylation substrate sites also resulted in deficient phosphorylation (data not shown). We next investigated whether deletion of the extracellular or cytoplasmic domain of FGFR2 can prevent colocalization. The 293T cells transiently transfected with CD44-v5 tag and FGFR2-myc tag plasmids were treated with PBS or FGF7 and stained with DAPI and a tag-specific antibody. Untransfected 293T cells were used as a negative control to confirm the absence of v5 and the detectable endogenous myc tag. Control experiments with no FGF7 treatment and WT full-length FGFR2 resulted in diffuse colocalization of FGFR2 with CD44. With FGF7 treatment, the two molecules aggregated to a single punctate dot per cell (Figure 6B) as previously observed (Figure 2, Figure S4, and Figure 5). In contrast, FGFR2 ΔE and FGFR2 ΔC did not demonstrate aggregated colocalization with CD44 even after FGF7 treatment (Figure 6B). These truncated proteins only diffusely colocalized with CD44 irrespective of FGF7 treatment, indicating that both the C and E domains are dispensable for diffusive association, but are required for ligand-induced complex formation (Figure 6B). The conformational change to an aggregated complex from the diffusive association might be important in the cross talk of FGFR2 with CD44. Immunoprecipitation (IP) revealed that associations between FGFR2 and CD44 require both the E and C domains, as deletion of either the E or C domain inhibited the association (Figure S8). These data suggest that cross regulation between FGFR2 and CD44 occurred at the posttranscriptional level via differential regulation of c-Myc and Sox2 at the transcriptional level (Figure 6C).

### Frequent overexpression of FGFR2 in tumorigenic primary GC cells

To understand whether FGFR2 expression *in situ* correlates with tumorigenic potential in xenograft models, freshly dissected GC tissues were implanted in nude mice. Out of 105 samples, 16 primary GC tissues grew into tangible tumors within 3 weeks while the remaining samples did not grow until 16 weeks. Immunohistochemistry (IHC) staining revealed FGFR2 expression in 35 out of 105 frozen primary GC tissues (Table 1). FGFR2 was positively correlated with tumorigenic potential (p < 0.01, Student's t-test). Among the 16 primary GC masses from which tumorigenic patient-derived gastric cancer xenograft (PDGCX) cell lines were derived, 14 primary GC masses (87.5%) highly expressed FGFR2 (FGFR2^+/hi^). Among nontumorigenic GC masses (n = 89), only 21 primary GC masses (23.6%) were FGFR2^+/hi^.

**Table 1:** FGFR2 expression in primary GC tissues correlates with tumorigenicity in nude mice

In xenograft	FGFR2^−/low^	FGFR2^+/hi^	Subtotal	P value
#Tumorigenic (n)	2	14	16	< .01
#Nontumorigenic (n)	68	21	89	< .001
Subtotal	70	35	105	na

Number of tumorigenic and non-tumorigenic patient-derived gastric carcinoma tissue specimens in the nude mouse xenograft assay according to FGFR2 expression patterns.

*FGFR2-/low or FGFR2+/hi, negative/low or postive/high expression in primary GC by *in situ* immunohistochmistry (IHC); #Tumor mass was formed (tumorgenic) or not formed (nontumorigenic) in tested 3 nude mice for each individual patient's specimen within 16 weeks post subcutaneous injection of approximately 0.2 cm3 GC tissue blocks; n, the number of patient-derived gasric carcinoma speciment belonging to the indicated category.

## DISCUSSION

Frequent deregulation of FGFR2 and CD44 observed in GC inspired an investigation of a possible cooperation between these molecules. Provided that CD44 is a bona fide GCSC marker [4] and that FGFR2 is the most commonly deregulated RTK pathway in GC, CD44^+^/FGFR^+^ GC cells could be a key subset dictating tumor initiation, suggesting some coordination between these two molecules. In accordance with this hypothesis, cell fractions of unmodified FGFR2^+^ or CD44^+^ cell grew faster, formed more tumor spheres *in vitro*, and established faster growing and larger tumors *in vivo*. Consistent with FACS results (Figure S1D, fraction of Y-axis >10^3^ fluorescence intensity), IF staining and confocal microscopy showed that most cells expressed CD44, but only a subset (∼20%) of CD44^+^ cells expressed FGFR2. Of this subset, only 5% expressed FGFR2 at high levels in SNU-16 GC cells. This result indicates that almost all FGFR2^+^ cells are in fact CD44^+^/FGFR2^+^ double positive, further implicating a possible interplay between these molecules (Figure 2, Figure S4, Figure 3). In contrast, the double negative control cells could not be enriched by FACS because of their extreme rarity, thus preventing a comparative study between the double positive and double negative fractions for tumor growth. Furthermore, IHC staining demonstrated frequent substantial FGFR2 overexpression in tumorigenic primary GC cells.

Though this study is first to describe the mechanistic role of FGF2 in CD44^+^ GCSC growth, our results are consistent with previous reports that FGFR2 can maintain breast tumor initiating cells [30] and that high FGFR-2 IIIc expression confers stem cell features to pancreatic ductal adenocarcinoma cancer cells [31]. This study also provides experimental evidence supporting the reciprocal regulation between CD44 and FGFR2, particularly via c-Myc at the transcriptional level and likely via Sox2 (as modeled in Figure 6C).

Notably, the coregulation of FGFR2 with CD44 may depend on the FGFR2 phosphorylation status and direct interaction of FGFR2 with a partner protein such as CD44. Multiple experiments clearly showed that FGF7 induced aggregated colocalization, forming an intermolecular transient signaling complex of FGFR2 with CD44 near perimembrane sites or within the cytoplasm (Figure 2C, Figure S4B, Figure 5D). These FGFR2 molecules were diffusely distributed when cells were not treated with FGF7 (Figure 2A, 2B, 2D). These unequivocal results suggest a direct or indirect interaction between these two molecules. It is likely that CD44 associates loosely with FGFR2 but tightly with p-FGFR2 as the association was induced by phosphorylation.

Similar to this study, FGFR2 homodimerization downregulated Nanog, Sox2, and Oct4 transcription in embryonic stem cells [32]. We further investigated some of these transcriptional factors that regulate FGFR2 or CD44 expression. The results indicate that while FGFR2 suppressed c-Myc and Sox2 transcription, c-Myc positively activates FGFR2 and Sox2 mRNA transcription. While the effect of c-Myc on FGFR2 regulation is clear, the effect of c-Myc on CD44 regulation could occur indirectly via FGFR2 (Figure 3B). Overexpression of c-Myc not only activates (or represses) c-Myc target genes but also globally amplifies transcription, reducing rate-limiting constraints for tumor growth [33]. In this regard, c-Myc may activate FGFR2 and repress CD44 at the transcriptional level. The presence of four copies of c-Myc binding DNA sequences on the FGFR2 promoter may account for this activation [34]. However the exact mechanism of c-Myc regulation of CD44 transcription cannot be concluded from this study. This inconclusiveness may be due to an oscillation of FGFR2 upon c-Myc KD. A recent study showed that Twist1 elevates FGFR2 expression and correlates with GC progression [35].

Our experiments provide the following evidence of stemness properties in FGFR2^+/hi^ GC cells. 1) Unmodified, sorted FGFR2^+^ cells supported tumor growth in mice. Well-known GCSC markers such as CD44^+^ or EPCAM^+^ fractions were employed as positive controls. SP^+^ was included as an internal negative control, validating the specificity and sensitivity of the assays. 2) FGFR2 depletion suppressed tumor growth *in vitro* and *in vivo*. Both stable FGFR2 KD prior to subcutaneous injection and induced FGFR2 KD by doxycycline during growth in mice using multiple independent shRNAs from independent experimental replicates showed consistent suppression. CD44 KD was used as a positive control. 3) Enforced FGFR2 expression supported tumor formation in mice in independent cell lines engineered to express FGFR2. 4) FGFR2 colocalized with CD44 and EpCAM in multiple experiments in two independent cell lines. 5) FGFR2^+/hi^ GC cells had enlarged cell volumes, a putative indicator of cells with stemness properties. 6) FGFR2 KD depleted CD44 and CD44 KD reduced FGFR2 in multiple independent experimental replicates using four different GC cell lines. 7). Activation of FGFR2 augmented CD44 and CD44 activation increased FGFR2. 8) Confocal microscopy showed an induced association of p-FGF7 with CD44 in multiple cell lines from multiple experiments. 9) Treatment with FGFR2 kinase inhibitor or FGFR2 NAB substantially decreased the CD44^+/hi^ GCSC fraction.

Several novel findings are of particular importance. 1) FGFR2 KD was accompanied by a decrease in CD44 and CD44 KD decreased FGFR2, indicating a reciprocal regulation between FGFR2 and CD44 via the key stemness factor c-Myc. Sox2 may participate in such reciprocal regulation. 2) In most cases, FGFR2-expressing cells had bigger cell volumes. These data collectively demonstrate that FGFR2 in the context of CD44 may constitute an essential regulatory circuit governing cancer stemness. In summary, FGFR2 and CD44 are coregulated via maintain GC stemness.

## MATERIALS AND METHODS

### Ethics statement

This study received Institutional Review Board (IRB) approval and includes the protocol number. Fresh human gastric carcinoma samples were obtained from surgically dissected tumors at Samsung Medical Center (SMC). Informed, written consent was acquired from the patients or their legal guardians. The protocol for experiments with human materials was approved by the IRB of SMC (IRB File No. 2010-08-159). For animal experiments, protocols for this study (No. 20100210001) were reviewed and approved by the Institutional Animal Care and Use Committee (IACUC) of Samsung Biomedical Research Institute (SBRI). SBRI is an Association for Assessment and Accreditation of Laboratory Animal Care International accredited facility. Experiments abided by the Institute of Laboratory Animal Resources guide and were approved by the IACUC of SBRI.

### Reagents

Antibodies, reagents, and sequences for primers and shRNAs are listed (Table S1, S2). Recombinant human keratinocyte growth factor/fibroblast growth factor 7 (KGF/FGF7) (R&D Systems, 251-KG-010/CF), hyaluronic acid (HA) (R&D Systems, GLR001), PD173074 (Sigma Aldrich, cat. # P2499), and FGFR2 neutralizing antibody (R&D Systems, cat. # MAB6843 clone 98739) were purchased from the indicated sources.

### Expression constructs, shRNA-mediated KD, IP, immunoblotting, IHC, IF, confocal microscopy and flow cytometry, tumor sphere assay, and tumor engraftment assay in mice

Standard protocols were used unless otherwise mentioned in the Supplemental Materials and Methods in Supplemental Documents.

## SUPPLEMENTARY MATERIALS FIGURES AND TABLES


