# Supplementary material for: A reciprocal regulatory circuit between CD44 and FGFR2 via c-myc controls gastric cancer cell growth
Source: Oncotarget. 2016 Apr 16;7(19):28670–83. doi: 10.18632/oncotarget.8764 (PMC5053754; doi:10.18632/oncotarget.8764)
Supplement: Supplementary file 1 [file oncotarget-07-28670-s001.pdf]

A reciprocal regulatory circuit between CD44 and FGFR2 via c-myc controls gastric cancer cell growth

SUPPLEMENTAL MATERIALS AND METHODS

Animal source, reagents, and tissue culture

Balb/c nude mice were purchased from Orient Bio Inc. (Gyeonggi, Korea). Non-obese diabetic/severe combined immunodeficient (NOD/LtSz-scid/IL2Rγ^{null}; referred to as NSG) mice were purchased from the Jackson Laboratory (Bar Harbor, ME) [1]. Newborn animals for transplantation experiments were obtained by inbreeding and were maintained under Specific-Pathogen-Free (SPF) conditions. Established and primary gastric cancer cells were cultured in RPMI containing 10% fetal calf serum (FCS) with antibiotics (R10) for routine maintenance unless otherwise mentioned. 293T cells were cultured in DMEM with 10% FCS and antibiotics (D10).

Expression constructs and shRNA-mediated knock down

Full length FGFR2IIIb (epithelial type transcript variant) was PCR-cloned into pcDNA3.1(+)/myc-His A (Invitrogen) using the indicated primer set (see Table S2). Mutant FGFR2 with a deleted extracellular domain (E) from a.a. 22 to 377 (R2ΔE) or a deleted intracellular cytoplasmic domain (C) from a.a. 399 to 821 (R2ΔC) were generated by PCR deletion methods using specific primer sets (Table S2). MKN45 GC cells that express CD44 but not FGFR2, and hence display weak tumorigenicity in mice, were transfected with FGFR2-myc wild type (WT) by AMAXA program L-029 and were selected with G418 (neomycin; 400 μg/m). In KD experiments, at least two different small hairpin RNA (shRNA) sets selective for each FGFR2 (five sets), CD44 (two sets), and c-Myc (five sets) were used for cloning into constitutive or doxycycline (Dox)-inducible vectors (see Table S2). Three sets of c-Myc siRNA (sets 6, 7, 8) were employed wherever necessary. The plasmid was transfected into the cells with the AMAXA kit using solution V and protocol O17. Conditional sub-cell lines in which FGFR2 or CD44 is subjected to depletion by Dox treatment were generated by the standard antibiotic selection protocol.

Screening a highly tumorigenic GC cell line in the xenograft condition

Three BALB/c nude mice per cell line were subcutaneously (s.c.) injected with 10,000 cells from each of the eight human GC cell lines: SNU-1, SNU-5, SNU-16, SNU-484, SNU-620, SNU-638, SNU-719, MKN45. Tumor size at 4 weeks post injection was measured. Where necessary, SNU-16 cells were stably labeled with firefly luciferase in order to monitor tumor growth and spreading real time *in situ*.

Confocal microscopy, immunofluorescence (IF) staining, and flow cytometry (FC)

Cells were smeared on the IF slide glass, dried quickly, fixed with 4% paraformaldehyde, permeabilized with 0.25 % Triton X-100 (where necessary), and incubated with 5 % (w/v) BSA in 1X PBS for 30 minutes at room temperature. The cells were then stained with diluted primary antibody (either dye non-conjugated or directly conjugated with FITC, PE, APC or PECy5), followed by incubation with the corresponding secondary antibody conjugated to Alexa Fluor® 568 (red) or Alexa Fluor® 488 (green) where necessary (see Table S1 for lists of antibodies). For confocal microscopy, the stained cells were mounted with Prolong Gold Antifade Reagent with DAPI (Invitrogen), and were examined using a Leica TCS SP5 spectral confocal system (LSM 710). For FC, cells in single cell suspension were incubated with the indicated dye conjugated or non-conjugated primary antibody. Where necessary, the indicated secondary antibody reactive to the non-conjugated primary antibody was used to label the cells. When necessary, the cell fractions positive for specific markers were then sorted by FACSAria (Beckton) and were subjected to secondary assays including tumor sphere, cell growth, real time quantitative PCR, or tumorigenesis.

Immunoprecipitation (IP) and western blotting

Cells were lysed in 1 ml of 1% Triton X-100, 150 mM NaCl, 1 mM EDTA, 50 mM Tris HCl pH 7.4 supplemented with 0.1% leupeptin and 0.01% phenylmethylsulfonyl fluoride (PMSF), and 200 nM sodium vanadate (NAV)/1 M sodium fluoride (NaF), where necessary. The lysates were cleared by centrifugation at 12,000 rpm for 5 min at 4°C and quantitated by Bradford method [2]. For IP, the lysate was precleared by protein A/G agarose and subjected to IP at 4°C overnight as previously described [3]. An equal amount of protein or equal volume of immune precipitate was separated on SDS-denaturing polyacrylamide gel, transferred to nylon membrane, and reacted to appropriate antibody.

Tumor sphere assay and patient-derived GC xenograft tumorigenic assay

To test xenograft tumorigenic assays of tumor samples from patient specimens, fresh GC tissues from 105 GC patients were minced and 0.2 cm^3 tissues were engrafted in nude mice (n ≥3 per each GC patient) for 12–16 weeks. Tumors were passaged *in vivo* to new mice from tumor-bearing mice without culturing *in vitro* until at least three passages were completed. Also, the indicated cell numbers of indicated cell lines were subcutaneously or orthotopically implanted in nude mice or NSG mice (wherever indicated) and tumor growth was determined by measuring diameters or luminescence (for the Luc-labeled cells). For *in vitro* cultures, tumor tissues were minced and cultured in flasks in RPMI 1640 supplemented with 10% FCS. For the tumor sphere assay, cells were plated at a density 100 cells per well in ultra low cluster 96 well plates (Costar, #3474) containing TeSR™ 2 media (STEMCELL, #05860), cultured for 14 days, and scored for size and number of spheres.

REFERENCES

1. Shultz LD, Lyons BL, Burzenski LM, et al. Human lymphoid and myeloid cell development in NOD/LtSz-scid IL2R gamma null mice engrafted with mobilized human hemopoietic stem cells. J Immunol 2005;174:6477-89.
2. Bradford MM. A rapid and sensitive method for the quantitation of microgram quantities of protein utilizing the principle of protein-dye binding. Anal Biochem 1976;72:248-54.
3. Kang MS, Lee EK, Soni V, et al. Roscovitine inhibits EBNA1 serine 393 phosphorylation, nuclear localization, transcription, and episome maintenance. J Virol 2011;85:2859-68.
4. Lee EK, Song KA, Chae JH, et al. GAGE12 mediates human gastric carcinoma growth and metastasis. Int J Cancer 2015;136:2284-92.
5. Nikitovic D, Kouvidi K, Karamanos NK, et al. The roles of hyaluronan/RHAMM/CD44 and their respective interactions along the insidious pathways of fibrosarcoma progression. Biomed Res Int 2013;2013:929531.

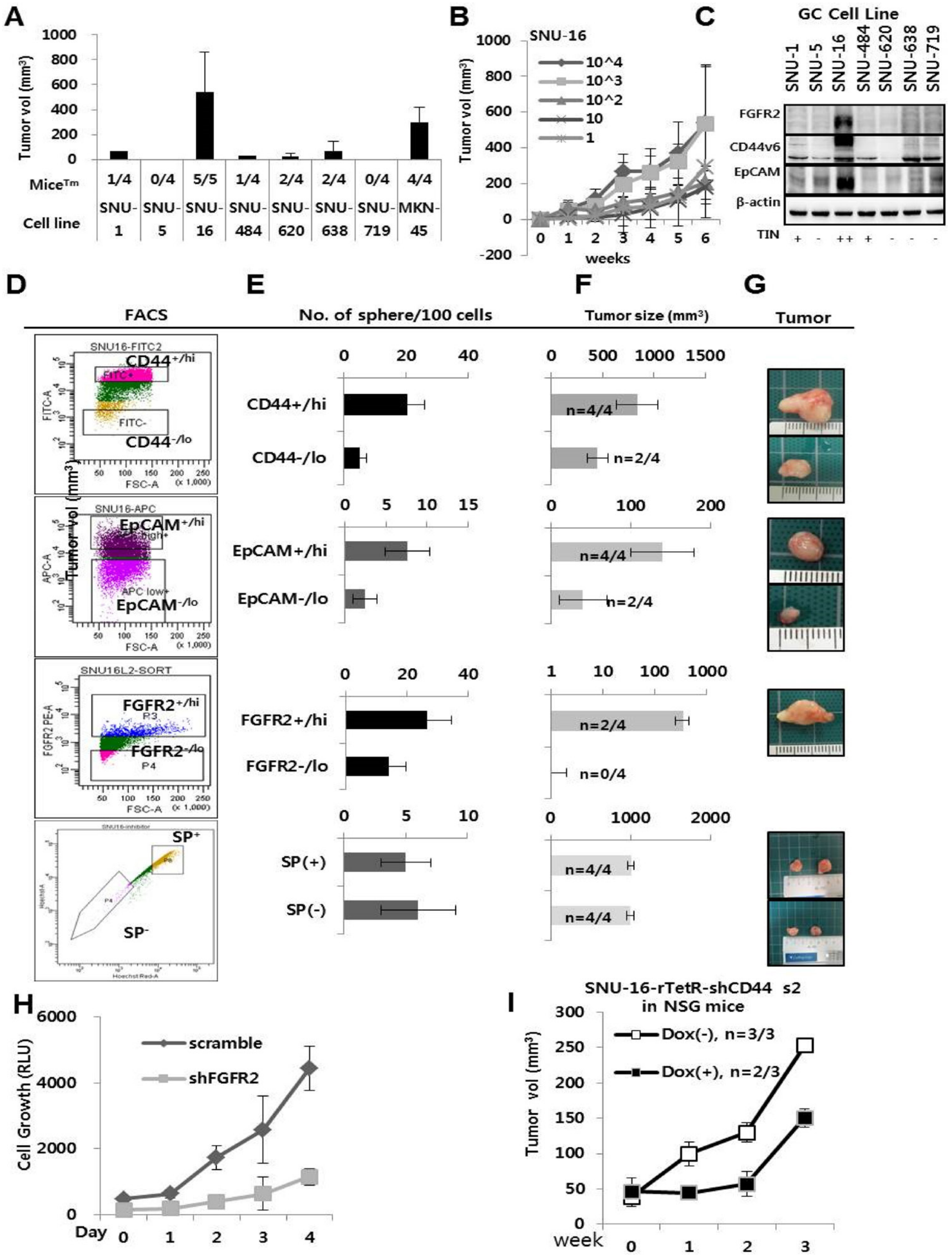

Supplementary Figure S1: Sorted FGFR2$^{+/hi}$, CD44$^{+/hi}$, or EpCAM$^{+/hi}$ fractions, but not side population (SP)$^{+}$ fractions, of gastric cancer (GC) cell lines supported tumor growth *in vitro* and *in vivo*. A. Tumorigenicity in nude mice (n = 4–5) by subcutaneous injection xenograft models. The number of tumor-bearing mice out of total mice injected as (Mice™/Total) upon implantation of one million cells of the indicated GC cell lines and the resultant tumor size (mm^3) are presented. SNU-16 and MKN45 showed high and modest tumorigenic potential (5/5 mice, tumor size average 538 mm^3; 4/4, tumor size 298 mm^3, respectively). **B.** Single cell-derived tumorigenicity of the SNU-16 GC cell line, indicating the presence of cancer stem cells. **C.** Expression of known gastric cancer stem cell (GCSS) markers (CD44, EpCAM) and FGFR2 in established GC cell lines. The highly tumorigenic SNU-16 cell line was strongly positive for CD44 and FGFR2. **D–G.** Unmodified, sorted CD44$^{+/hi}$, EpCAM$^{+/hi}$, FGFR2$^{+/hi}$, but not side population (SP)$^{+}$, of SNU-16 cells (D) formed more tumor spheres 14 days after plating (E) and formed larger tumors after s.c. injection of an equal number of indicated cell fractions in nude mice (n = 4) (F) relative to control cells expressing none or low levels of the indicated markers. SP^{+} is defined as a flow cytometry fraction that became low or faint in Hoechst 33342 upon verapamil treatment, a reversing agent of a multidrug resistant protein. The representative tumor masses are presented (G). Well-known GCSC markers CD44^{+} or EPCAM^{+} fractions were employed as positive controls and SP^{+} was an internal negative control, validating the specificity and sensitivity of the assay. **H.** FGFR2 KD mediated by shRNA set (s) 1 suppressed cell growth. **I.** Dox-induced CD44 knockdown (KD) *in vivo* suppressed tumor growth in NSG mice.

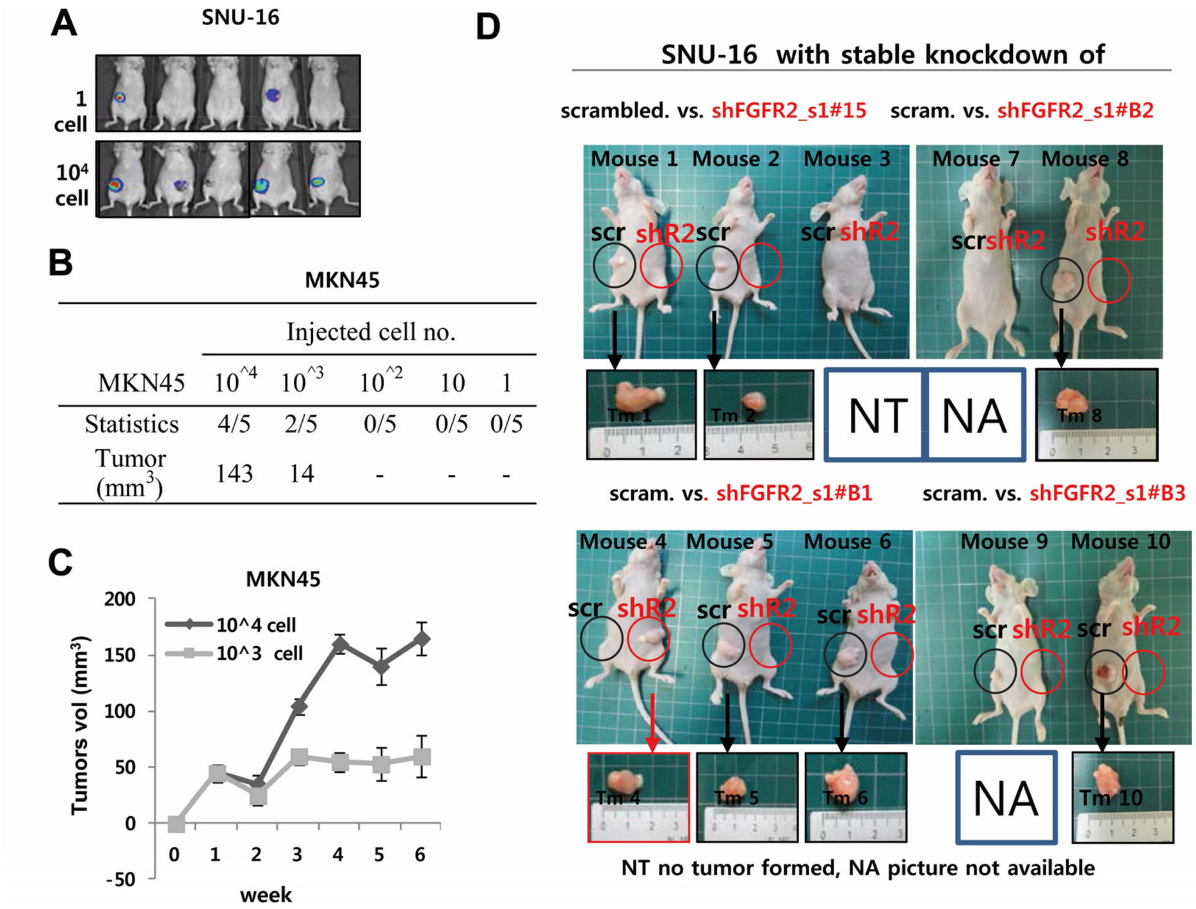

Supplementary Figure S2: Additional data supporting Figure S1 and Figure 1. A. Formation of tumor mass from 1 or 10^4 cells from the highly tumorigenic SNU-16 cell in nude mice. Tumor growth was monitored *in situ* by labeling the cells with luciferase (See SI for detail). **B–C.** Moderate tumorigenic potential of MKN45 requiring at least 1000 cells in nude mice (C) and size of tumor mass upon injection of indicated cell number (D). In detail, the growth of SNU-16 cells was monitored in real time by stably labeling the cells with firefly luciferase as previously reported [4]. The luminescence intensity of luciferase labeled SNU-16 was 3.78 RLU/cell, sufficient to monitor the bioluminescence in mice. Luciferase-labeled SNU-16 cells were comparable to the mother SNU-16 cells in their ability to form tumors *in vivo* in a dose- and time-dependent manner. All mice (n = 5) formed s.c. tumors when injected with 100, 1,000, and 10,000 cells. In 1- and 10-cell injected groups, 2/5 mice formed tumors of considerable size with viable bioluminescence. Strikingly, mice from the 1 cell-injected group developed tumors with an average size of 2.93 ± 0.94 cm^3 at 6 weeks post injection, which was approximately half the average size observed in mice injected with 10,000 cells (5.38 ± 3.28 cm^3) (Figure S1). The SNU-16 cell line expressed CD44, FGFR2, and EpCAM at considerable levels. **D.** Knockdown (KD) of FGFR2 in SNU-16 suppressed tumor growth in mice. Shown are the gross images of Figure 1A.

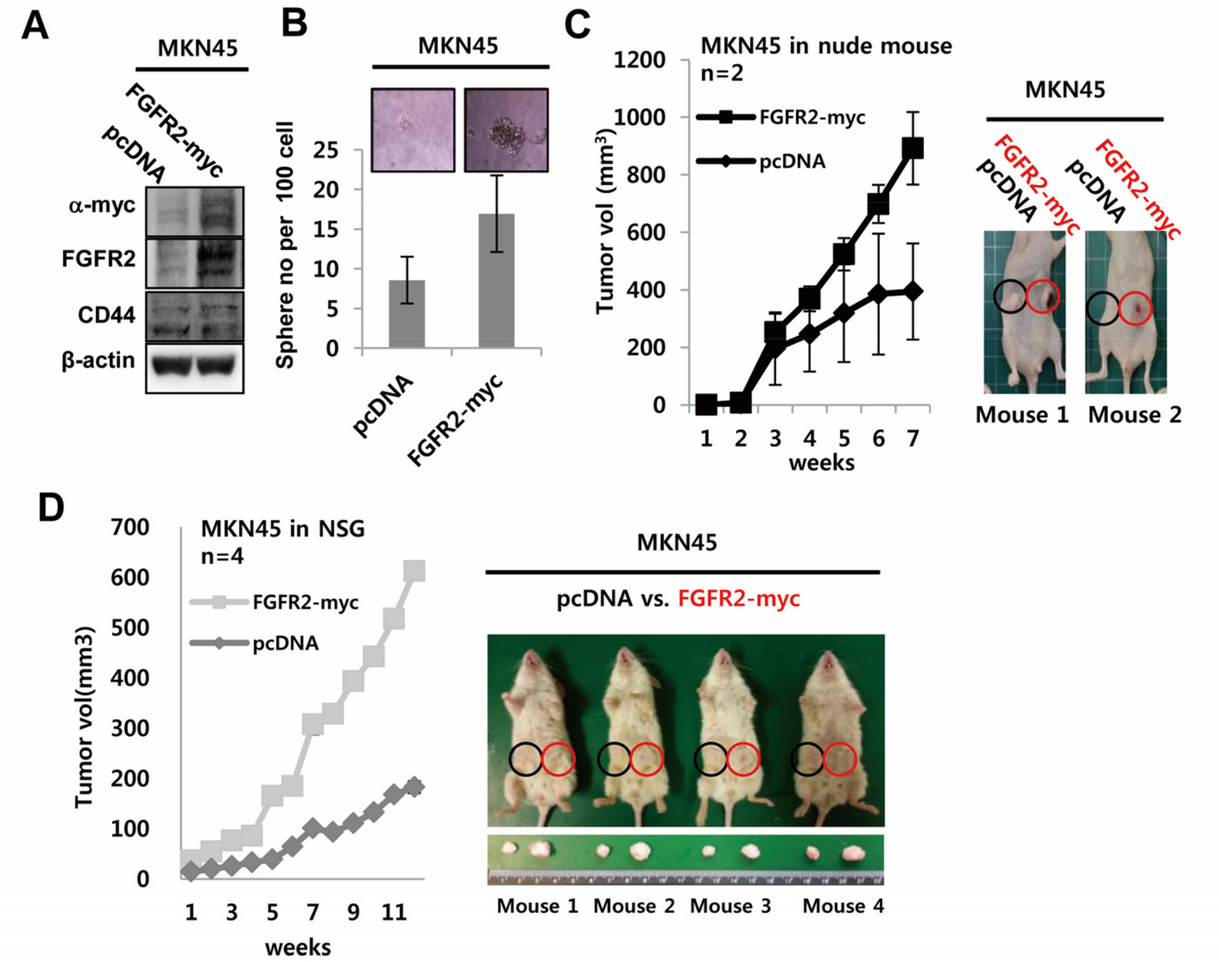

Supplementary Figure S3: Enforced FGFR2 expression supported the formation of tumor spheres and tumors. **A.** Stable expression of FGFR2 or pcDNA vector in MKN45 cells inherently expressing CD44 (left). **B.** Enhanced formation of tumor spheres by enforced FGFR2 expression relative to a vector control as evidenced by sphere culture assays for 14 days with 100 cells in stem cell media ($P < 0.0001$) (right). **C–D.** FGFR2-expressing MKN45 formed bigger and more rapidly growing tumors than vector-expressing cells in nude mice (C) and NSG mice (D) upon s.c. injection (left). Gross finding of the tumor mass formed is shown (right).

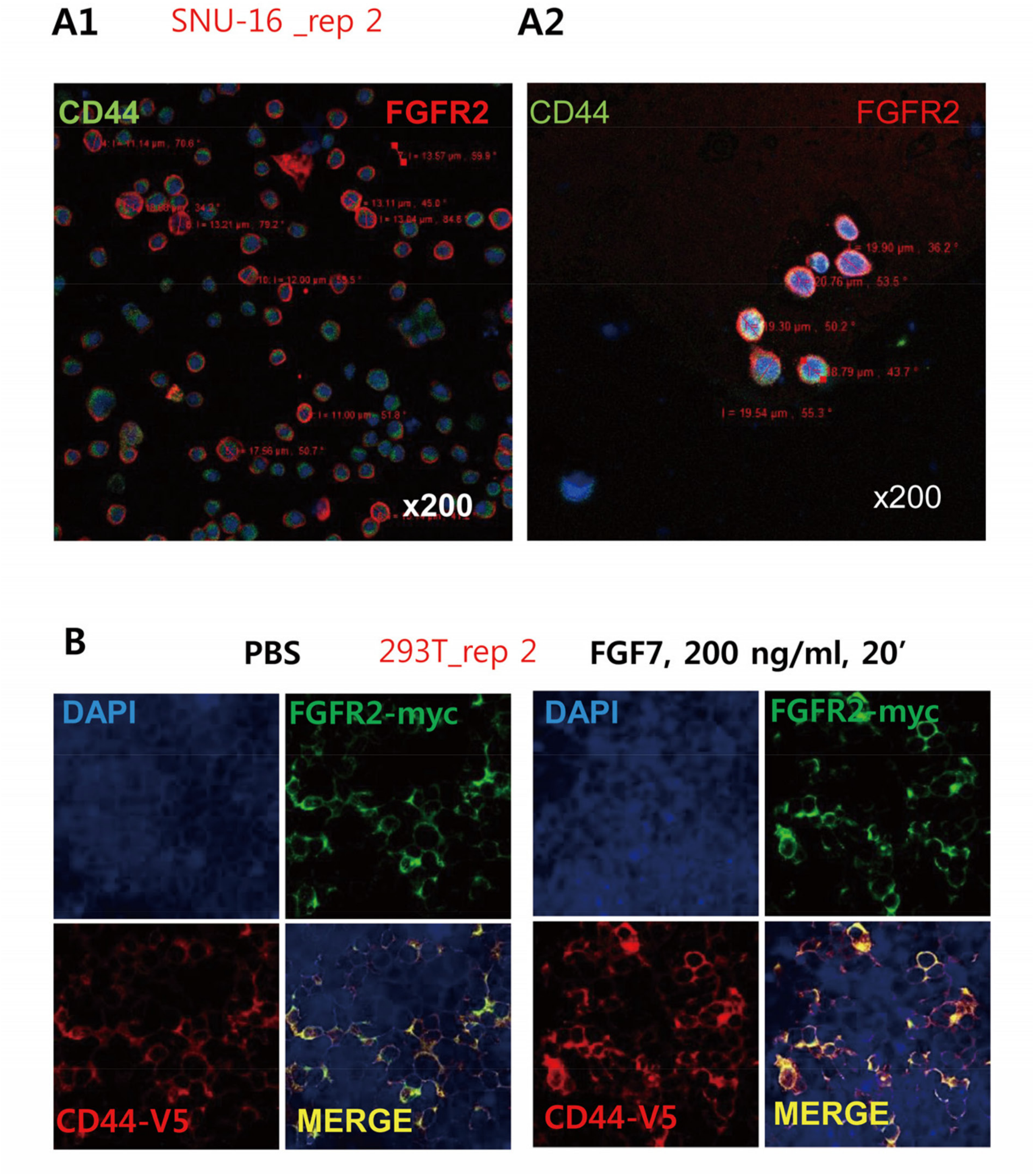

Supplementary Figure S4: Additional data supporting Figure 2. Confocal images of immunofluorescence staining with FGFR2, CD44, and EpCAM in cultured cells. A1. FGFR2-expressing cells had enlarged cell volumes. **A2.** Increased cell diameter in CD44/FGFR2-highly positive cells collected from two independent experiments (A1 and A2). **B.** Induced colocalization of FGFR2 with CD44 upon FGF7 treatment, a natural ligand for FGFR2 in an independent 293T cell line. The 293T cells were transiently transfected with indicated vectors expressing CD44-V5 and FGFR2-myc. Transfected cells were treated with FGF7 (200 ng/ml) for 20 min, subjected to immunofluorescence staining, and visualized by confocal microscopy. See more experimental replicates in Figure 4, Figure 7B, and Figure S5.

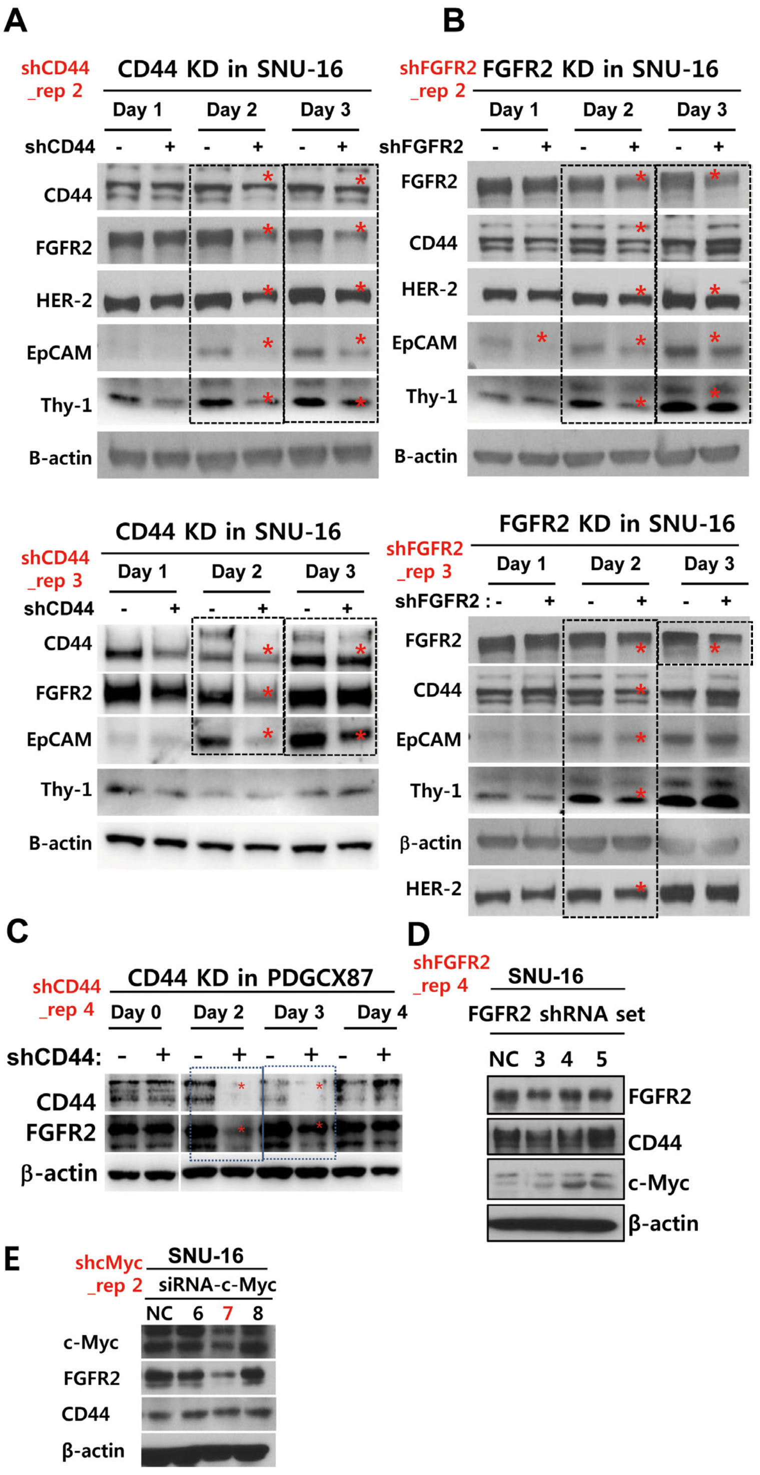

Supplementary Figure S5: Experimental replicates supporting Figure 3. Transient knockdown (KD) of FGFR2 depletes CD44 and CD44 depletion decreases FGFR2 via c-Myc. A. An shRNA set 1-mediated transient KD of CD44 decreased FGFR2, HER2, EpCAM, and Thy1 (CD90) in multiple independent experiments. **B.** An shRNA set 1-mediated transient KD of FGFR2 decreased CD44 (albeit weakly) and EpCAM (albeit transiently), but neither HER2 nor Thy-1. * denotes a decrease in mRNA level at the indicated days following KD. **C.** An shRNA set 1-mediated transient KD of CD44 decreased FGFR2 in a patient-derived GC xenograft-87th primary cell line *in vitro* (PDGCX87). **D.** Two shRNA sets (set 3 and 4)-mediated FGFR2 KD consistently decreased CD44, albeit to varying degrees. **E.** The siRNA set 7-mediated KD of c-Myc decreased FGFR2. * denotes a decrease in mRNA level at the indicated days following KD.

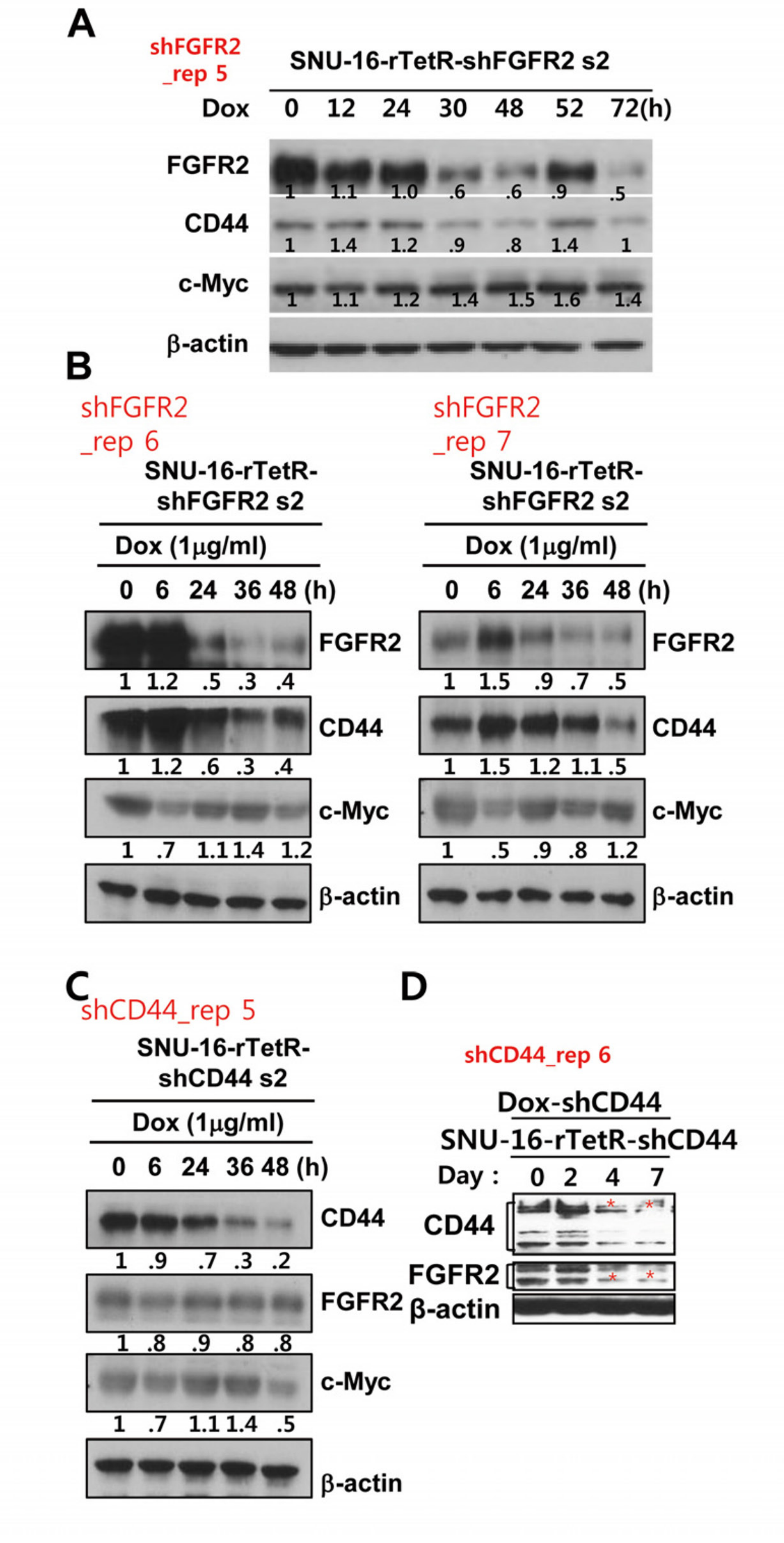

Supplementary Figure S6: Additional replicates supporting Figure 3 and Figure S5. Conditional knockdown (KD) of FGFR2 depletes CD44 and CD44 KD reduces FGFR2 via c-Myc. A–B. Following FGFR2 KD induced by Dox, an FGFR2 decrease was relatively well correlated with CD44 but inversely correlated with c-Myc levels, despite occasional oscillation at the late stage of KD, indicative of the presence of feedback regulation. Numbers indicate expression normalized to beta-actin. **C–D.** Induced CD44 KD by Dox treatment for 2 days (C) or for up to 7 days (D) was accompanied by decreased FGFR2 protein levels in the SNU-16-TetR-shCD44 set 2 SNU-16 line.

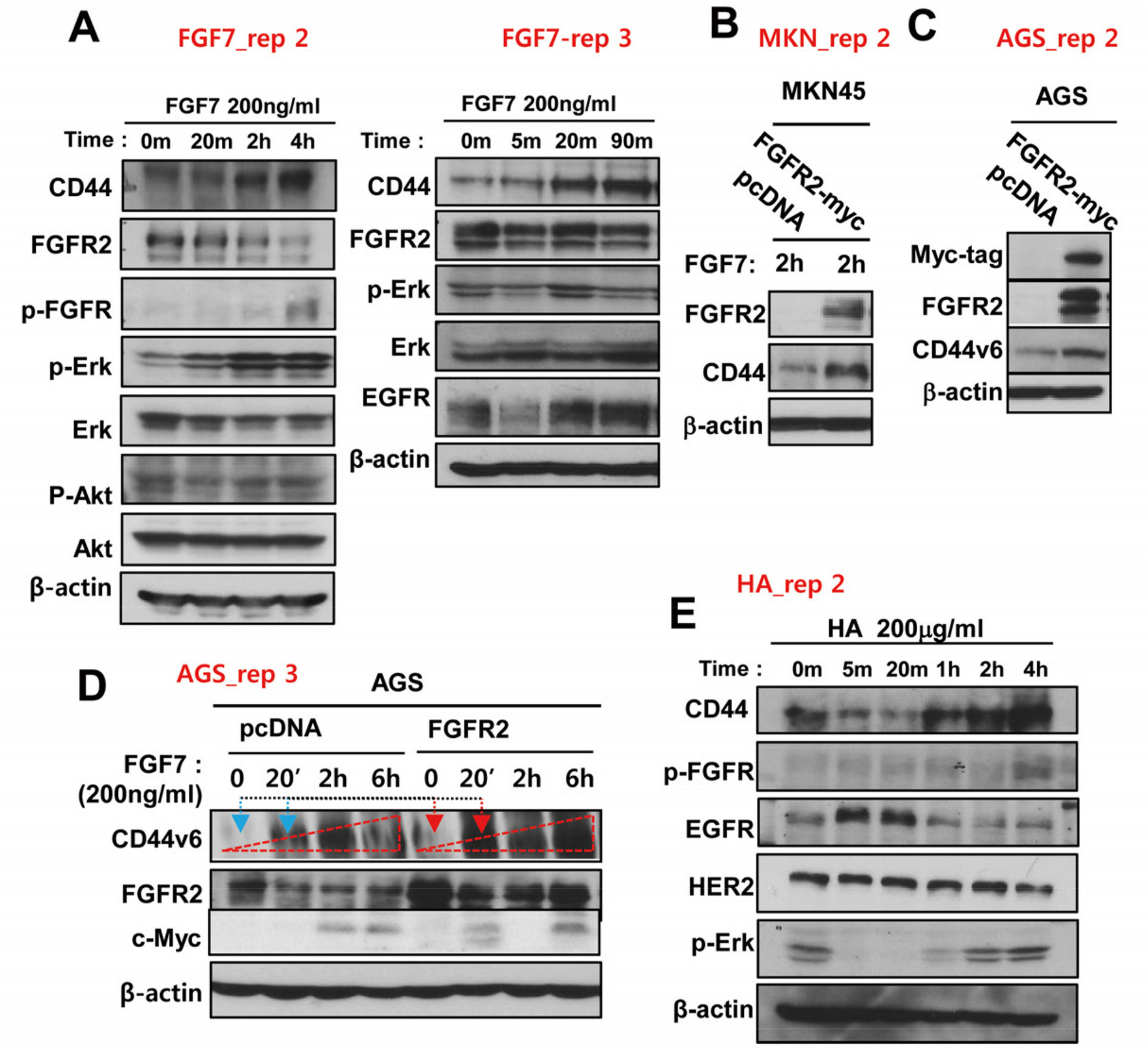

Supplementary Figure S7: Additional data supporting Figure 4. FGFR2 activation augments CD44 signaling and CD44 activation enhances FGFR2 signaling. A. FGFR2 activation by FGF7 increased CD44 and caused concomitant transient phosphorylation of FGFR2 (delayed) and ERK. **B.** Transfection of FGFR2 into the FGFR2$^{-/low}$, CD44^{low} MKN45 GC cell line increased CD44 in response to FGF7. **C.** Transfection of FGFR2-myc into the FGFR2$^{-/low}$, CD44^{low} AGS GC cell line and subsequent activation by FGF7 increased CD44. **D.** Transfection of FGFR2 into the FGFR2$^{-/low}$, CD44^{low} AGS GC cell line and subsequent activation by FGF7 drastically increased CD44 (see ascending dotted triangle) with concomitant decreases in FGFR2 because of receptor internalization. CD44 increased in FGFR2-transfected cells (red arrow) relative to pcDNA-transfected cells (red arrow). **E.** CD44 activation by hyaluronic acid (HA) results in an initial decrease and subsequent increase in CD44, which was colinear to p-FGFR2 and p-ERK, downstream of both CD44 and FGFR2 pathways [5]. HA treatment over 12 hr reduced CD44 levels (data not shown).

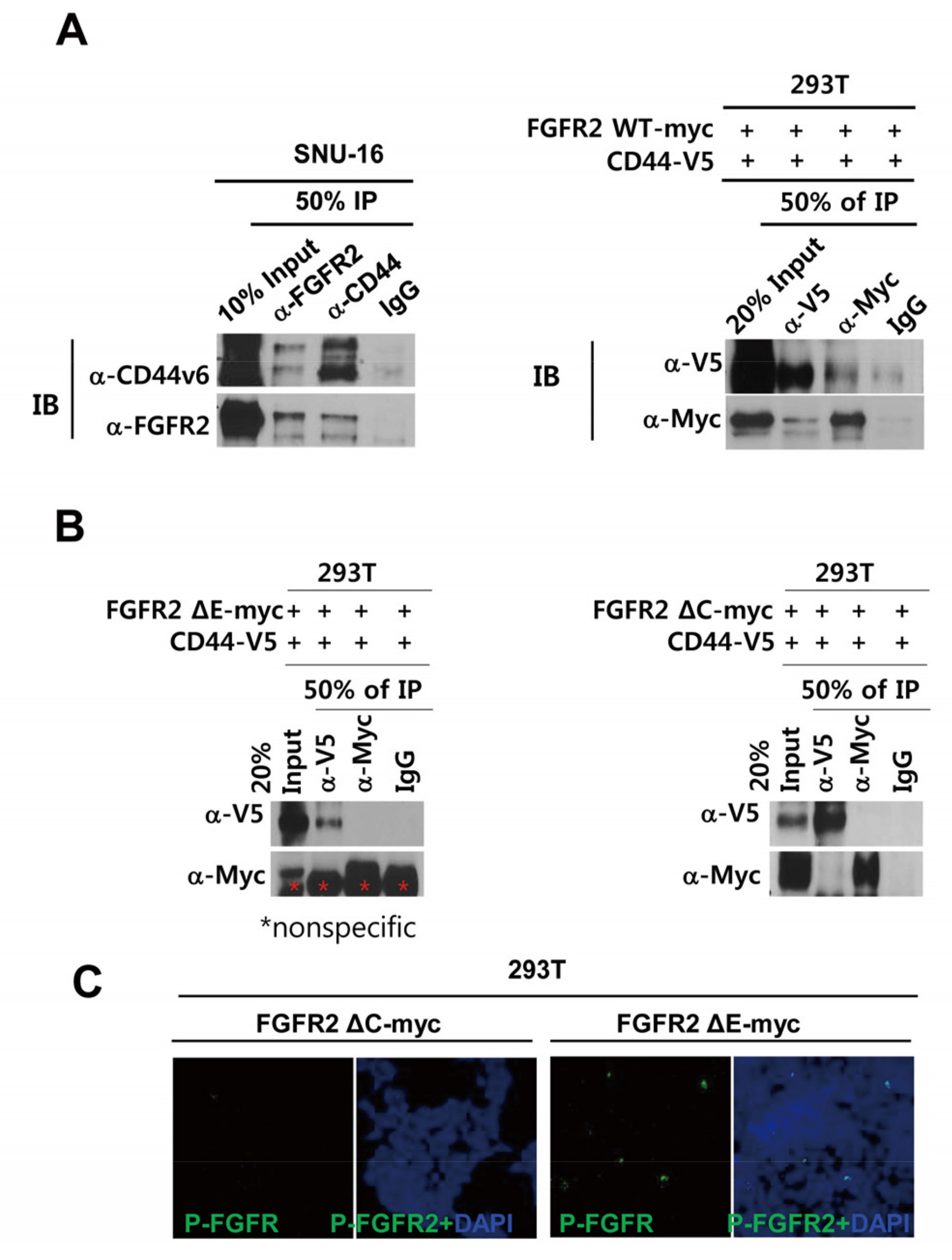

Supplementary Figure S8: Association of FGFR2 with CD44 requires both extracellular (E) and cytoplasmic (C) domains. A. Co-IPs of CD44 and FGFR2 in SNU-16 (left) and 293T cells (right) transiently transfected with constructs expressing FGFR2 WT-myc and CD44-V5. Less than 0.5% of total FGFR2 interacted with CD44 (compare an intensity (*) of 50% immune precipitates with 10–20% input levels). **B.** Co-IPs between CD44-V5 and FGFR2ΔE-myc or FGFR2ΔC-myc. Full length CD44-V5 interacted with both FGFR2ΔE-myc and FGFR2ΔC-myc, indicating that both E and C domain of FGFR2 are necessary for an association with CD44 in cells. **C.** Lack of phosphorylated FGFR2 (p-FGFR2) in response to FGF7 (200 ng/ml) in 293 T cells transiently transfected with FGFR2ΔE-myc or FGFR2ΔC-myc.

Supplementary Table S1: List of antibodies used in this study

Name	Alias	Clone name	Manufacture	Cat #
CD44v6	Pgp-1, H-CAM, Ly-24	2F10	R&D Systems	BBA13
c-MYC. Myc tag		9E10	Santa Cruz	sc-40
EpCAM	ESA, EpCAM	323/A3	Santa Cruz	sc-73491
h/mCD44 FITC	Pgp-1, H-CAM, Ly-24	IM7	BD Pharmingen	553133
hCD326 APC	ESA, EpCAM	HEA125	Miltenibiotech	130-091-254
hCD90 PECy5	THY1	5E10	BD Bioscience	555597
HER2		Rabbit polyclonal	Cell signaling	2242
hFGFR2	dye non conjugated	98725	R&D Systems	FAB684A
hFGFR2		3F8	Abcam	ab119327
LGR5		Rabbit polyclonal	SIGMA	HPA012530
p-FGFR2		Rabbit polyclonal	Cell signaling	3471
p-FGFR2(Y653/Y654)		Rabbit polyclonal	R&D Systems	AF3285
TetR		9G9	Clontech	631131
THY1	CD90	Rabbit polyclonal	Cell signaling	9798
V5 tag		mouse monoclonal	Invitrogen	46-0705
Anti-mouse Ab-Alexa 488		NA	Invitrogen	a2120216
Anti-mouse Ab-Alexa 568		NA	Invitrogen	a11004

Supplementary Table S2: List of primers used in this study

Category	Gene		Sequence	Remarks (Vendor)
shRNA	CD44	set 1	CCGGTATGCAATGTGCTACTGATTGTCTCGAG<br>ACAATCAGTAGCACATTGCATTTTTTG	Addgene
Dox_shRNA	CD44	set 2	CCGGGACAGAAAGCCAAGTGGACTCAACGGAGA<br>CTCGAGTCTCCGTTGAGTCCACTTGGCTTTCTGTC<br>TTTTTG	Genolution
shRNA	FGFR2	set 1	CCGGTGAAGATGTTGAAAGATGATGCCACAGAG<br>CTCGAGCTCTGTGGCATCATCTTTCAACATCTTCA<br>TTTTTG	Origene
	FGFR2	set 3	CCGGACGACCAAGAAGCCAGACTTCAGCAGCCA<br>CTCGAGTGGCTGCTGAAGTCTGGCTTCTTGGTCGT<br>TTTTTG	Addgene
	FGFR2	set 4	CCGGTGAAGATGTTGAAAGATGATGCCACAGAG<br>CTCGAGCTCTGTGGCATCATCTTTCAACATCTTCA<br>TTTTTG	Addgene
	FGFR2	set 5	CCGGGGAAGGAGTTTAAGCAGGAGCATCGCATT<br>CTCGAGAATGCGATGCTCCTGCTTAAACTCCTTCC<br>TTTTTG	Addgene
Dox_shRNA	FGFR2	set 2	CCGGGGAAGGAGTTTAAGCAGGAGCATCGCATT<br>CTCGAGAATGCGATGCTCCTGCTTAAACTCCTTCC<br>TTTTTG	Genolution
shRNA	c-Myc	set 1	CCGGCCATAATGTAAACTGCCTCAACTCGAG<br>TTGAGGCAGTTTACATTATGGTTTTTG	TRCN0000039638
	c-Myc	set 2	CCGGCAGTTGAAACACAAACTTGAACTCGAG<br>TTCAAGTTTGTGTTTCAACTGTTTTTG	TRCN0000039640
	c-Myc	set 3	CCGGCAGGAACTATGACCTCGACTACTCGAG<br>TAGTCGAGGTCATAGTTCCTGTTTTTG	TRCN0000039641
	c-Myc	set 4	CCGGCCTGAGACAGATCAGCAACAACTCGAG<br>TTGTTGCTGATCTGTCTCAGGTTTTTG	TRCN0000039642
	c-Myc	set 5	CCGGAACTATGACCTCGACTACGACTCGAG<br>TCGTAGTCGAGGTCATAGTTCTTTTTG	TRCN0000010389
siRNA	c-Myc	set 6	GACAGUGUCAGAGUCCUGA	Bioneer
	c-Myc	set 7	AUCUAACUCGCUGUAGUAA	Bioneer
	c-Myc	set 8	CGUAAGGAAAAGUAAGGAA	Bioneer
Cloning	CD44	F	5-CCGGAATTCATGGACAAGTTTTGGTGGCAC	
		R	5-CCTCCCCTCGAGCACCCCAATCTTCATGTCCAC	
	FGFR2IIIb	F	CGGGATCCGCATGGTCAGCTGGGGTCGTTCATCT	
		R	CCTCCCCTCGAGTGTTTTAACACTGCCGTTTATGTGT	
	FGFR2IIIbΔE	F	ATGATAGCCATTTACTGCATAGGGGTCTTC	
		R	GCGGATCCGAGCTCGCTACCAAGCTTA	
	FGFR2IIIbΔC	F	CTCGAGTCTAGAGGGCCCTTCGAACAAAAACTC	
		R	GCACAGGATGACTGTTACCACCATACAGGCGAT	
qPCR	FGFR2	F	ATGGATAAGCCAGCCAACTG	
		R	TGCTTGAACGTTGGTCTCTG	
	CD44	F	AAGGTGGAGCAAACACAACC	
		R	ACTGCAATGCAAACTGCAAG	
	EpCAM	F	CTGCCAAATGTTTGGTGATG	
		R	AAAGCCCATCATTGTTCTGG	
	Thy1(CD90)	F	GGACTGAGATCCCAGAACCA	
		R	ACGAAGGCTCTGGTCCACTA	
	Sox2	F	GCACATGAACGGCTGGAGCAACG	
		R	TGCTGCGAGTAGGACATGCTGTAGG	
	c-myc	F	AATGAAAAGGCCCCCAAGGTAGTTATCC	
		R	GTCGTTTCCGCAACAAGTCCTCTTC	

Direction 5'–>3', The underlined denotes gene-specific seqeunce, F, forward; R, reverse.
